# Dyads' Perceptions: Recruiting Persons Living With Dementia and Caregivers in a Clinical Trial

**DOI:** 10.1093/geroni/igab046.807

**Published:** 2021-12-17

**Authors:** Justine Sefcik, Darina Petrovsky, Glenna Brewster, Junxin Li, Nalaka Gooneratne, Nancy Hodgson, Miranda McPhillips

**Affiliations:** 1 Drexel University, College of Nursing and Health Professions, Philadelphia, Pennsylvania, United States; 2 Rutgers University, Philadelphia, Pennsylvania, United States; 3 Nell Hodgson Woodruff School of Nursing Emory University, Atlanta, Georgia, United States; 4 Johns Hopkins University, Baltimore, Maryland, United States; 5 University of Pennsylvania School of Medicine, Philadelphia, Pennsylvania, United States; 6 University of Pennsylvania, School of Nursing, Philadelphia, Pennsylvania, United States; 7 University of Pennsylvania, University of Pennsylvania, Pennsylvania, United States

## Abstract

Recruiting persons living with dementia (PLWD) and their caregivers (dyads) into research is challenging and costly. The purpose of this study was to better understand factors that influence dyads decisions to enroll in a clinical trial. We used Ajzen’s Theory of Planned Behavior (TPB) to develop a qualitative interview guide and analyze the data with a directed content analysis. We conducted semi-structured telephone interviews with 12 PLWD and 9 caregivers who all enrolled in one clinical trial. Aligning with the TPB we found the following positively influenced enrollment: 1) wanting to learn, in-person meetings with knowledgeable staff, and the money always helps (attitudes toward joining); 2) to support another person (perceived norm); and 3) easy to participate (perceived behavioral control). Flexible scheduling and the study taking place in the home was comfortable and convenient for participants. Findings can inform future recruitment efforts and research studies.

